# Successful Non-operative Management of Metacarpophalangeal Collateral Ligament and Sagittal Band Rupture of the Long Finger: A Case Report

**DOI:** 10.7759/cureus.26231

**Published:** 2022-06-23

**Authors:** Brian T Bueno, Tiffany A Smith, Michael J Lotito, Joseph V Phelan, Robert E Epstein, Brian M Katt, David Kirschenbaum

**Affiliations:** 1 Orthopaedic Surgery, Rutgers Robert Wood Johnson Medical School, New Brunswick, USA; 2 Radiology, Rutgers Robert Wood Johnson Medical School, New Brunswick, USA

**Keywords:** finger, management, sagittal band, collateral ligament, metacarpophalangeal

## Abstract

The metacarpophalangeal (MCP) joint is surrounded by various structures critical to its stability and function. Though the ligamentous injury to the digits is common, rupture of the metacarpophalangeal collateral ligament and a sagittal band of the same finger is not well represented in the literature. We report a chronic case of a concurrent metacarpophalangeal collateral ligament and sagittal band injury. Though surgery would have been the most appropriate treatment soon after the injury, restrictions on elective procedures due to the COVID-19 pandemic precluded surgical treatment. The patient was alternatively treated with buddy tape, and a close follow-up was done. This is the first reported case of a concurrent metacarpophalangeal collateral ligament, and sagittal band injury successfully treated using nonoperative management.

## Introduction

The sagittal band and collateral ligament of the metacarpophalangeal (MCP) joint are often subject to traumatic injury. Though isolated injuries to both structures have been well described in the literature, concurrent injury to both structures in the same finger is rare and minimally reported [1,2]. We present a case in which a patient suffered a radial collateral ligament and sagittal band injury of the long finger, received no initial treatment, and was successfully treated without surgery.

## Case presentation

The patient is a 63-year-old, right-hand-dominant male who fell down three steps onto his non-dominant hand and wrist. The patient immediately felt pain in the left long MCP joint, went to an emergency department, and was evaluated. Radiographs were reported negative, and the patient was discharged without a splint. After following up with his primary care physician, the patient returned to work after two days despite continued pain.

**Figure 1:**
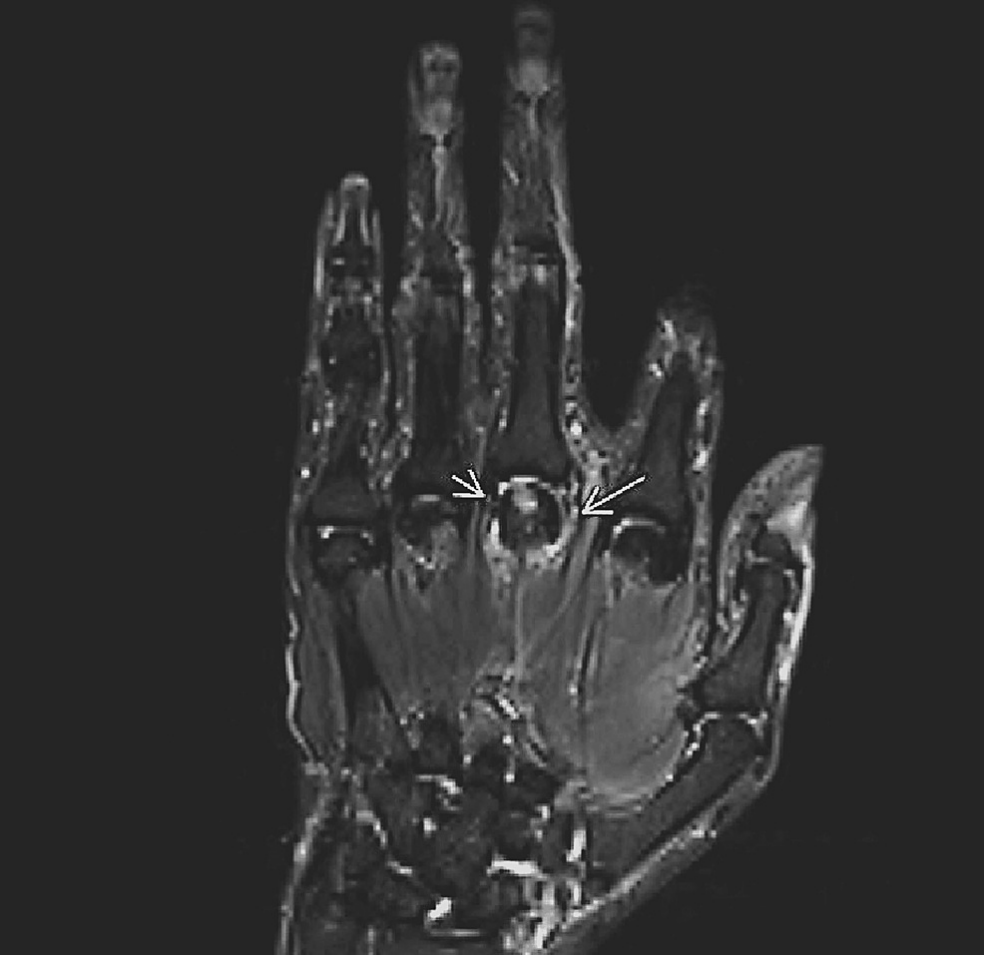
Coronal STIR 3T image of metacarpophalangeal joint Coronal STIR 3T image acquired through the long finger MCP. A long arrow demarcates the avulsed proximal aspect of the RCL from the MC head without a Stener lesion. The short arrow demarcates the low signal, taught, intact UCL. Note the MCP fluid, regional edema, and underlying cystic change in the MC head. STIR- short TI inversion recovery; MCP- metacarpophalangeal; RCL- radial collateral ligament; UCL- ulnar collateral ligament

**Figure 2:**
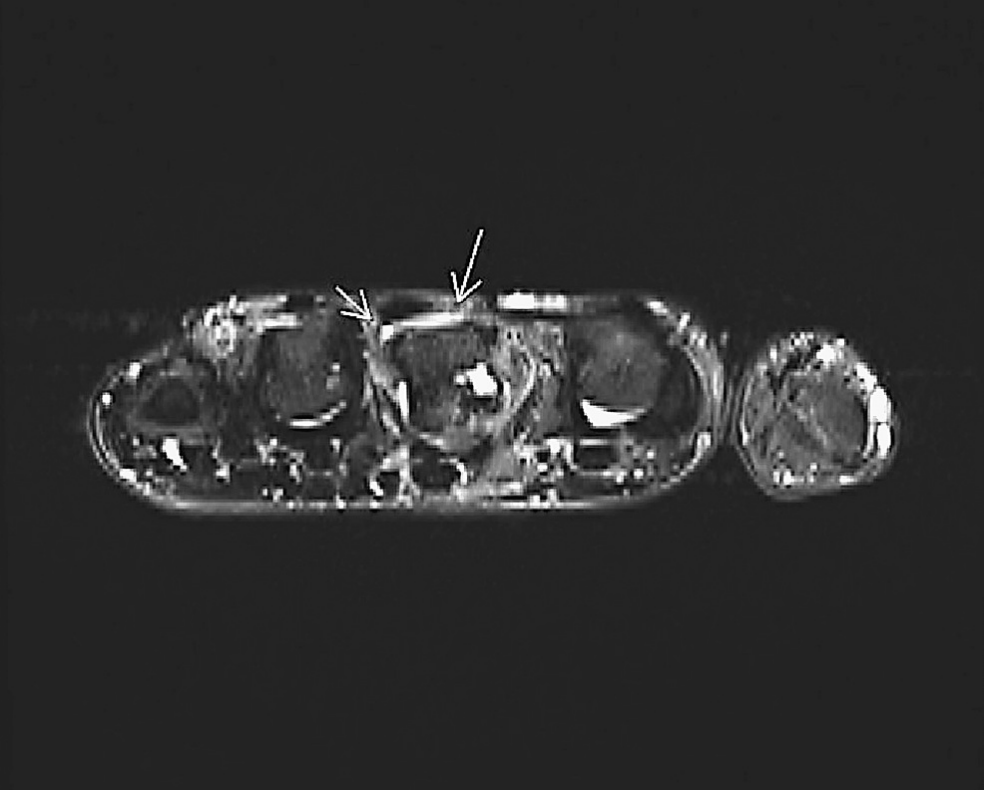
Axial T2 weighted 3T image of the metacarpophalangeal joint Axial T2 3T weighted image acquired at the long finger MC head level. Long arrow demarcates torn irregular, indistinct radial sagittal band. A short arrow demarcates the lower signal, taught, intact sagittal ulnar band. Note that the ring finger extensor tendon is aligned correctly, but the long finger extensor tendon is 50% ulnarly subluxated.

Due to delays related to COVID-19, the patient was seen in an outpatient orthopedic office three months post-injury. On examination, the patient had swelling and tenderness on the dorsal aspect of the radial side of the long MCP joint. There was also obvious ulnar deviation of the long finger with ulnar subluxation of the extensor tendon. The patient had no restrictions in flexion of any digits but had extension weakness and a 15-degree extensor lag in the affected finger. Due to the chronicity of the problem and instability of the radial collateral ligament along with ulnar drift, an MRI was suggested to confirm the pathology. The MRI demonstrated a rupture of the sagittal radial band of the long finger MCP joint, a proximal rupture of the radial collateral ligament, and extensor tendon subluxation (Figure 1, 2).

Due to COVID-19 restrictions on elective surgical cases, the patient was sent home with buddy tape and instructed to tape the index and long fingers together in the interim. The patient was instructed to follow up in one month to reassess his symptoms and accessibility of performing surgery.

At one month follow-up, the patient continued to display ulnar deviation and difficulty when fully extending the long finger. The patient was amenable to surgical reconstruction of the sagittal band and radial collateral ligament and relocation of the extensor tendon. The surgery was scheduled when the moratorium on elective surgery was lifted. However, during a telemedicine follow-up to discuss preparation for surgery, the patient expressed disinterest in moving forward with surgical intervention, as he was concerned with the surgical risks during the COVID-19 pandemic. Additionally, he reported having no pain or functional deficits in his hand.

**Figure 3:**
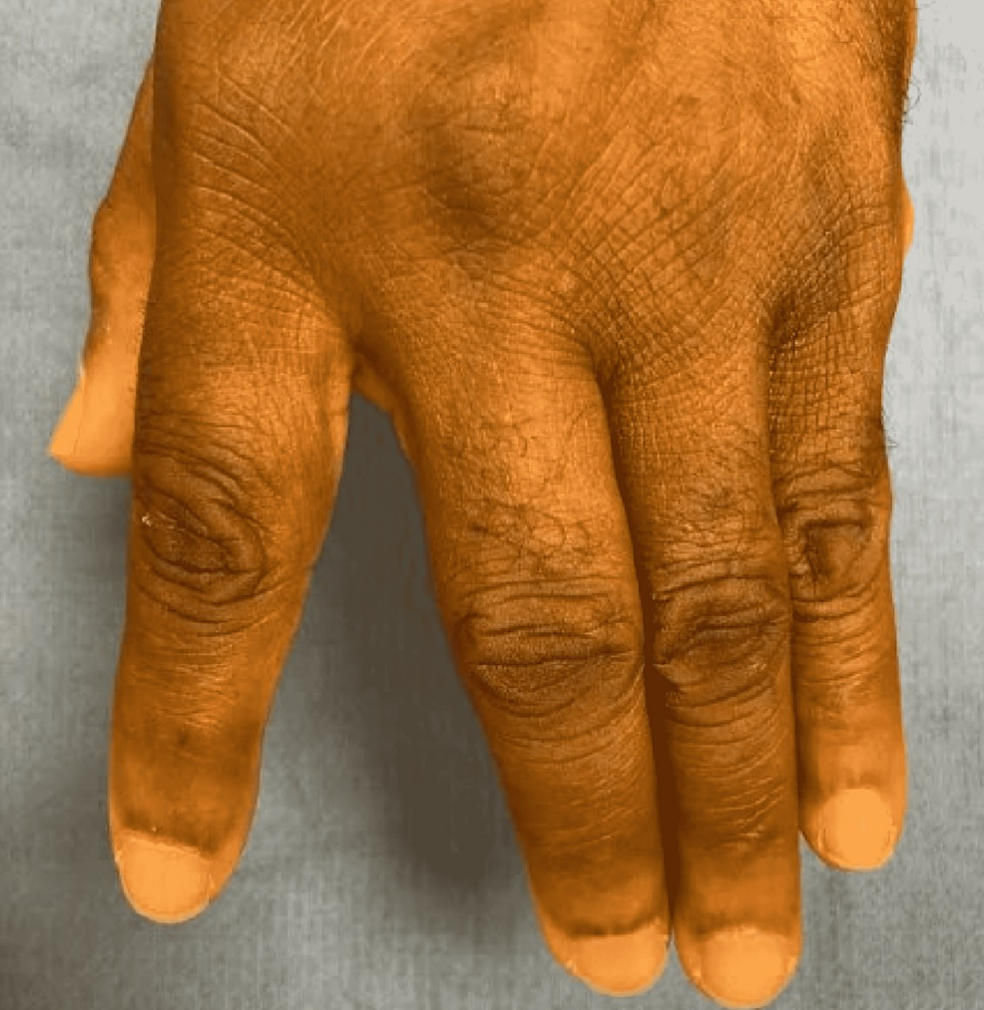
Photograph of dorsal aspect of hand with fingers in extension A photograph of the dorsal aspect of the patient is a left hand with fingers in extension at a two-year follow-up. Thirty-five degrees of ulnar drift is noted at the long MCP joint. MCP- metacarpophalangeal.

**Figure 4:**
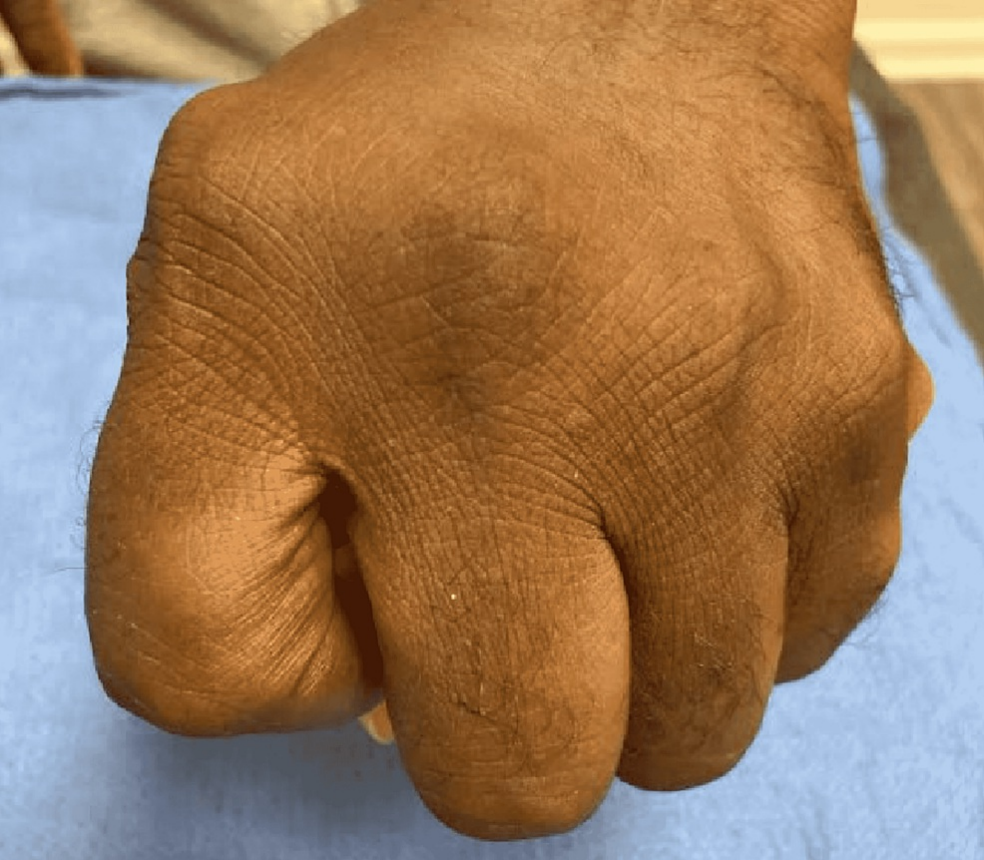
Photograph of dorsal aspect of hand with fingers in flexion Photograph the dorsal aspect of the patient's hand with fingers in flexion at a two-year follow-up. Ulnar drift is maintained at the long MCP joint. MCP- metacarpophalangeal.

At our request, the patient followed up two years after the injury. He had no pain or difficulty using the hand and discontinued buddy taping. He used the full tape time for three months and then sporadically after that. On exam, he continued to have 35 degrees of ulnar drift with extensor digitorum communis (EDC) subluxation without complete dislocation in the valley between the long and ring metacarpal heads. (Figures 3, 4). He was able to make a full fist, but the 15-degree extensor lag persisted (Figure 4). Stressing the radial collateral ligament (RCL) in slight flexion revealed an endpoint at 45 degrees without pain compared with 20 degrees on the contralateral side. Despite not having surgery, overall hand function was very good.

## Discussion

Acute MCP collateral ligament ruptures & management 

Acute injury to the collateral ligament of the metacarpophalangeal (MCP) joint can occur in any of the digits of the hand. The injury commonly results after a fall on the hand or while trying to grasp an object. Upon physical examination, tenderness and instability while testing the MCP joint in flexion are defining features of this injury [3]. Literature on this injury indicates conservative treatment unless there is gross instability of the joint [3,4].

Pedrazzini et al. described an MCP radial collateral ligament rupture of the long finger with a similar lesion of the deep, transverse metacarpal ligament and gross instability of the joint [5]. Nonetheless, Ishizuki et al. note that MCP collateral ligament ruptures of non-thumb digits are rare [6]. The authors suggest that the low prevalence of ruptures of non-thumb digits may arise from frequent misdiagnosis as a sprain. This assertion may be warranted, given that a subsequent case series reported 38 cases of non-thumb digit ruptures [1].

The literature currently supports a wide array of management types for acute collateral ligament injuries of the MCP joint of the fingers, ranging from interdigital buddy taping to surgical intervention. Gaston et al. created a treatment-guiding grading system for injuries to the index finger MCP joint [7]. This grading schema utilizes the laxity of the MCP joint to classify the injury accurately. Grade 1 was defined as tenderness to palpation over the RCL but no measured laxity compared with the contralateral digit. Grade 2 was defined as laxity more significant than the contralateral digit with a definite, firm endpoint. Grade 3 was defined as increased laxity compared to the contralateral digit but having no discernable endpoint [7]. Patients with grade 1 and grade 2 MCP joint injuries were successfully managed nonoperatively through buddy taping, splinting, casting, and strengthening exercises.

Conversely, surgical intervention was recommended for grade 3 injuries caught early. No discernible endpoint to ulnar stress was noted initially in the patient above, indicating a grade 3 injury. Surgical intervention might have been indicated for our patient if the recommendations of Gaston et al. were generalized to injuries of the long finger MCP joint. However, restrictions to elective surgery as a result of the COVID-19 pandemic precluded this option, and our patient decided against reconstruction even after operating rooms were reopened. Ultimately, our patient's pain resolved, and although ulnar drift persisted, the function was excellent.

Sagittal band ruptures & management 

Sagittal band ruptures were first described in 1957, displaying swelling and thickening of the soft tissue surrounding the affected joint [8]. The varying tendon and capsule rupture prevalence led Gladden et al. to propose a Type I-IV classification system [8]. Specifically, Type I injuries present without evidence of ruptures, Type II injuries present with superficial ruptures, Type III injuries present with ruptures extending into the tendon/capsule, and Type IV injuries present with ruptures extending into the joint space.

Posner & Ambrose later reported the injury in six professional athletes, and nearly all patients attained a full range of motion postoperatively [9]. The authors noted that accompanying extensor tendon subluxation resulted in more severe cases, a finding supported by a subsequent case series [10,11]. Our patient presented similarly with dynamic subluxation of the extensor tendon into the groove between the third and fourth digits with ulnar deviation of the long finger.

As seen in the current patient, sagittal band ruptures typically present in the long digit with a painful and swollen MP joint. This injury affects the long finger due to the length and relatively weak attachment to the extensor hood [12]. Furthermore, MP extension is uncomfortable if initiated from a flexed position and may be impossible when extensor dislocation is present. Crepitus may also result from tendon subluxation, creating a pseudo-trigger finger and often resulting in misdiagnosis as a trigger finger [13]. Our patient presented with a 15-degree lag to full extension and showed weakness when a counterforce was applied but did not exhibit crepitus. 

Kleinheinz & Adams built off of Gladden's Type I-IV injury classification to suggest that non-surgical management can be effective for most type I and type II injuries and many type III injuries. In contrast, type IV injuries typically require surgery [13]. Successful non-surgical management has been reported in the literature: Ritts et al. utilized dorsal splinting in two patients with sagittal band ruptures, Catalano et al. treated ten patients with a digital extension orthosis referred to as a sagittal band bridge, and Wu et al. found nonoperative management to be a successful approach in injuries presenting within three weeks [11,14,15]. Our patient presented with a type IV rupture based on the presence of extensor tendon subluxation at his initial visit, three months following his injury. Based on the Kleinheinz & Adams classification system, surgery was therefore indicated. Another recent revision of sagittal band injury classification by Sivakumar et al. classified our patient's injury as Type IV: chronic injury (>6 weeks) with extensor tendon subluxation or dislocation [16]. These authors suggest that Type IV injuries are unlikely to be successfully managed non-operatively. However, the present patient experienced full symptomatic relief and recovered hand function despite only receiving nonoperative treatment.

Concurrent MCP collateral ligament & sagittal band ruptures 

Concurrent MCP collateral ligament and sagittal band ruptures of the same digit are rarely reported in the literature. Of the few reported cases, most consist of concurrent injuries with a Stener-like lesion. Stener-like lesions of the finger involve the ruptured end of the collateral ligament being trapped by the open window of the injured sagittal band. This often leaves the finger in a deviated position.

The most documented affected site of a Stener-like lesion of the finger is the radial side of the little finger, which appears in the abducted position on examination. Ishizuki et al. described the clinical presentation of these injuries as an abducted little finger, inability to adduct the little finger, and instability of the MP joint. The five patients Ishizuki et al. discussed with Stener-like lesions of the small finger were all treated with surgical intervention. Dennison et al. reported a complete MCP radial collateral ligament avulsion with sagittal band rupture of the small finger, which was successfully treated surgically [2].

The patient presented in this case presented with a rupture of the sagittal radial band of the long finger MCP joint and a proximally ruptured radial collateral ligament. However, the injury pattern did not mimic a Stener-like lesion, as there was no trapping of the ruptured radial collateral ligament by the open window of the ruptured sagittal band.

Ishizuki et al. presented a similar case to ours in which a patient sustained one concurrent injury of the collateral ligament and sagittal band in the long finger [1]. Despite both cases involving the same structures, they differ by treatment. The patient presented by Ishizuki et al. was treated with surgical intervention, whereas our patient was initially not treated and subsequently used buddy tape. The patient described by Ishizuki et al. was reported to have a slight residual limitation of the joint six months post-operation. In contrast, the patient in this case who underwent nonoperative management had no functional limitations. Had there been no pandemic, our patient would likely have undergone reconstruction of the sagittal band and RCL. Operating room closures made surgery impossible, and even after the restriction was lifted, some patients were hesitant to move forward with elective surgery. Although the literature does support non-surgical treatment for acute injuries, nonoperative treatment for subacute and chronic cases is not a clear standard.

As a result of the COVID-19 pandemic, we had the opportunity to evaluate a single case with an RCL and sagittal band rupture associated with EDC subluxation and report excellent function two years after non-surgical management. Based on this case report, we cannot recommend nonoperative treatment for everyone, especially those with frank dislocation of the extensor tendon in the valley with no ability to extend the finger actively. However, nonoperative treatment will result in good function in some subacute and chronic circumstances despite a cosmetic ulnar drift deformity. There is no reason not to consider splinting and buddy tape or surgery for acute injuries.

## Conclusions

Concurrent injury to the radial collateral ligament and the sagittal band is an exceedingly rare and scantly reported injury. Perhaps underrepresented due to its misdiagnosis in the clinical setting, we hope to increase awareness of the potential for such an injury in the long finger. Furthermore, we describe the first reported case using non-surgical treatment to manage this chronic injury successfully.
